# Primate-specific miR-603 is implicated in the risk and pathogenesis of Alzheimer's disease

**DOI:** 10.18632/aging.100887

**Published:** 2016-02-08

**Authors:** Chi Zhang, Jie Lu, Bing Liu, Qinghua Cui, Yun Wang

**Affiliations:** ^1^ Neuroscience Research Institute and Department of Neurobiology, Key Laboratory for Neuroscience of Ministry of Education, National Health and Family Planning Commission, School of Basic Medical Sciences, Peking University Health Science Center, Beijing, 100191, China; ^2^ Department of Biomedical Informatics, School of Basic Medical Sciences, Peking University Health Science Center, Beijing, 100191, China; ^3^ PKU-IDG/McGovern Institute for Brain Research, Peking University, Beijing, 100871, China; ^4^ Brainnetome Center, National Laboratory of Pattern Recognition, Institute of Automation, Chinese Academy of Sciences, Beijing, 100190, China

**Keywords:** miR-603, single nucleotide polymorphism (SNP), Alzheimer's disease risk, pathogenesis, biogenesis

## Abstract

Alzheimer's disease (AD) is a serious neurodegenerative disease, and microRNAs (miRNAs) have been linked to its pathogenesis. miR-603, a novel primate-specific miRNA and an intronic miRNA of a human brain highly expressed gene KIAA1217, is implicated in the risk and pathogenesis of AD. The rs11014002 single nucleotide polymorphism (SNP) (C/U), which locates in miR-603 precursor (pre-miR-603), exhibits a protective effect towards AD risk. Additionally, the rs11014002 SNP promotes the biogenesis of mature miR-603. miR-603 downregulates LRPAP1 mRNA and protein levels through directly binding the 3′ untranslated region (3′UTR) of LRPAP1. Moreover, miR-603 increases LRP1 protein expression. LRPAP1 and LRP1, playing opposite roles, are involved in Aβ clearance and pathogenesis of AD. Strikingly, miR-603 exhibits a relatively higher expression and there is a loss of a negative correlation between miR-603 and LRPAP1/RND1 mRNA levels in the hippocampi of patients with AD. In addition, miR-603 directly downregulates a key neuronal apoptotic component-E2F1, and prevents HeLa cells from undergoing H_2_O_2_-induced apoptosis. This work suggests that miR-603 may be a novel AD-relevant miRNA and that its rs11014002 SNP may serve as a protective factor against AD.

## INTRODUCTION

Alzheimer's disease (AD) is characterized by a progressive loss of episodic memory and other cognitive functions and is a type of neurodegenerative disorder. Historically, AD has been chiefly defined by its pathological signature, which includes β-amyloid deposits in the form of extracellular amyloid β (Aβ) plaques and hyperphosphorylated Tau aggregates in the form of intracellular neurofibrillary tangles (NFTs) [1].

MicroRNAs (miRNAs) are 18–25 nucleotide long, non-coding RNAs that are processed by Drosha and Dicer in the nucleus and cytoplasm, respectively [2]. Typically, miRNAs result in translational repression by guiding the miRNA-induced silencing complex (miRISC) to the 3′ untranslated regions (3′UTRs) of mRNA targets [3]. Recently, numerous studies have indicated that miRNAs are implicated in a diverse array of brain functions, including development, cognition, and synaptic plasticity [4]. Moreover, several miRNAs have been found to be related to AD pathogenesis [5] and to affect the expression or function of AD-relevant molecules such as amyloid precursor protein (APP) [6], β-site amyloid precursor protein cleaving enzyme 1 (BACE1) [7, 8] or Tau [9],[10].

miR-603 was first discovered in human colorectal cells [11] and is a novel, primate-specific, intronic miRNA [12] whose host gene KIAA1217 is highly expressed in the brain [13]. KIAA1217 (Gene ID: 56243) is located at 10p12.31 and has been reported to be involved in the etiology of lumbar disc herniation [14] and genetic susceptibility of intervertebral disc degeneration [15]. Since the expression profiles of intronic miRNAs are frequently correlated with the expression profiles of corresponding host genes [16], miR-603 may also be expressed in the brain. Furthermore, primate-specific or human-specific properties of lncRNAs and miRNAs have shown potentially relevant to evolution of unique human neural properties and functions [17, 18]. In addition, recent studies revealed that miR-603 is involved in the tumorigenesis of central nervous system [19, 20]. Based on these, miR-603 is suggested to be associated with brain function. By analyzing the Alzheimer's Disease Neuroimaging Initiative (ADNI) database, we found that a single nucleotide poly-morphism (SNP), rs11014002, in miR-603 precursor (pre-miR-603) that is associated with a reduced risk of AD and probable later onset of mild cognitive impairment (MCI). We further demonstrated that miR-603 regulates several AD-related molecules. Strikingly, through studies on hippocampal samples of patients with AD, we observed that miR-603 exhibits a relatively higher expression and there is a loss of a negative correlation between miR-603 and LRPAP1/RND1 mRNA levels in the hippocampi of patients with AD. Taken together, these results suggest that miR-603 may be involved in AD pathogenesis and that the rs11014002 SNP may serve as a protective factor against AD.

## RESULTS

### The rs11014002 SNP carriers exhibit reduced risk of AD

**Figure 1:**
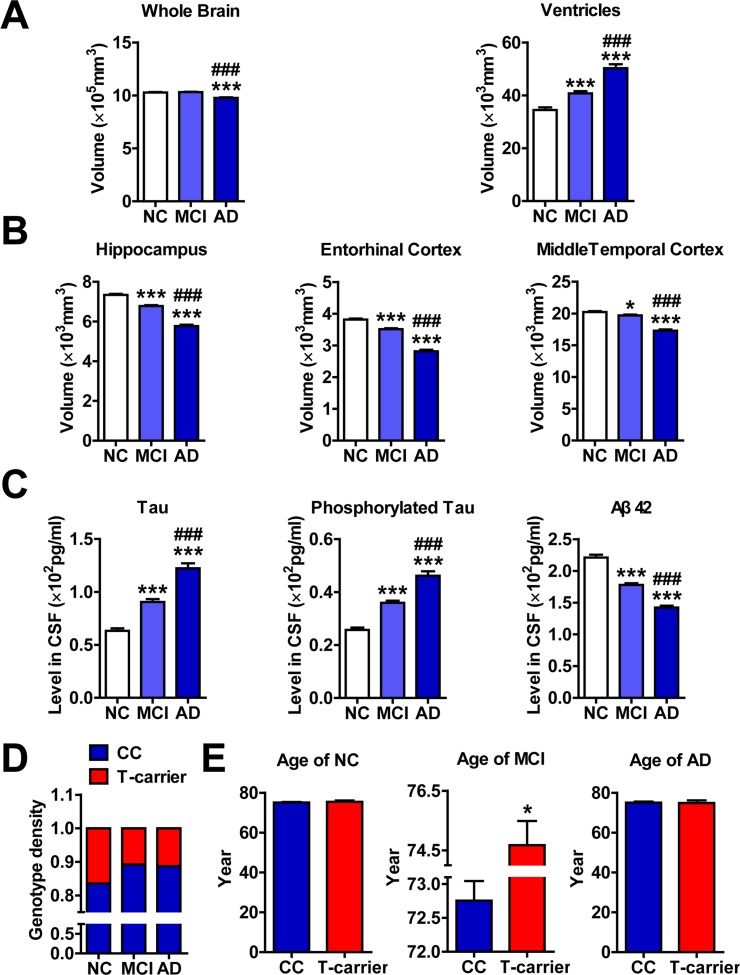
The rs11014002 SNP carriers exhibit reduced risk of AD (**A**), (**B**) and (**C**) Differential analysis of volumes of whole brain, ventricle, hippocampus, entorhinal cortex, and middle temporal cortex and levels of Tau, phosphorylated Tau and Aβ 42 in the cerebrospinal fluid (CSF) of cognitively normal elderly controls (NC), subjects with mild cognitive impairment (MCI) and subjects with Alzheimer's disease (AD) (n = 365, 772 and 301 for NC, MCI and AD subjects, respectively). One-way ANOVA was used for analysis. *P < 0.05, **P < 0.01, ***P < 0.001 versus the NC subjects; ^###^*P* < 0.001 versus the MCI subjects. (**D**) Genotype density among the NC, MCI and AD subjects; chi-square test was used for the analysis. (**E**) Differential analysis of ages of NC, MCI and AD subjects with the CC genotype and T-carrier genotype (n= 305 and 60 in the NC group; n= 689 and 83 in the MCI group; and n= 267 and 34 in the AD group for CC and T-carrier subjects, respectively). *P < 0.05, versus CC as determined by unpaired two-tailed Student's *t*-tests.

Our ADNI data analysis was consistent with previous studies [21] in that the whole brains of subjects with MCI and AD are progressively atrophic and the ventricles are enlarged (Figure 1A). Additionally, the hippocampi, entorhinal cortices and middle temporal cortices are increasingly atrophic (Figure 1B). Furthermore, the levels of Tau and phosphorylated Tau in the CSF are increased, whereas the levels of Aβ 42 are decreased (Figure 1C).

Based on the rs11014002 SNP (C/U), the genotypes of miR-603 were classified into two types: the CC genotype (wildtype) and the T-carrier genotype (CT or TT). We first revealed that the distributions of the two genotypes between NC, MCI and AD subjects are significantly different (Figure 1D; Table 1). Additionally, the T-carrier genotype exhibits a modest protective effect towards MCI risk (OR = 0.612, *p* =0.005, chi-square test) and AD risk (OR = 0.647, *p* =0.036, chi-square test). More intriguingly, although no significant difference in the average age between the CC genotype and T-carrier genotype was observed in NC and AD groups, the significantly higher average age of MCI subjects with the T-carrier genotype (CC: 72.7±7.6; T-carrier: 74.7±7.4) might suggest later onset of MCI (Figure 1E; Table 2).

**Table 1:** Genotypes and phenotypes of subjects from the ADNI database included in our study

Subject	CC		T-carrier		Total		
NC	305	(83.56%)	60	(16.44%)	365	(25.4%)	*P=0.02036
MCI	689	(89.25%)	83	(10.75%)	772	(53.7%)	
AD	267	(88.70%)	34	(11.30%)	301	(20.9%)	
Total	1261	(87.69%)	177	(12.31%)	1438	(100.0%)	

**Table 2:** Demographics of subjects from the ADNI database included in our study

Subject	Genotype	Sample size	Age	Gender (M:F)	*APOE* (ε4+:ε4−)
NC	CC	305	75.1±5.6	158:147	78:227
	T-carrier	60	75.4±5.2	33:27	18:42
		365	*P*=0.659	*P*=0.755	*P*=0.581
MCI	CC	689	72.7±7.6	408:281	359:330
	T-carrier	83	74.7±7.4	55:28	33:49
		772	*P*=0.028	*P*=0.263	*P*=0.056
AD	CC	267	75.1±7.9	145:122	172:95
	T-carrier	34	74.9±7.7	20:14	24:10
		301	*P*=0.884	*P*=0.752	*P*=0.603

ADNI: Alzheimer's Disease Neuroimaging Initiative.

NC: Normal control.

MCI: Mild cognitive impairment.

AD: Alzheimer's disease.

The significance of the above results was not a result of differences in sex, age, or *APOE* genotype (Table 2). Notably, although more males are included in ADNI studies, no significant difference in male-to-female ratios was observed among all groups (Table 2). Collectively, we reported that the rs11014002 SNP carriers exhibit reduced risk of AD and the rs11014002 SNP may serve as a protective factor against AD.

### The rs11014002 SNP promotes miR-603 biogenesis

According to recent studies, SNPs in pri-miRNA and pre-miRNA can affect miRNA maturation efficiency and therefore affect disease risk [22]. We hypothesized that the potential “biogenetic efficiency-related rs11014002 SNP” results in differential expression of miR-603, such that different phenotypes are produced.

**Figure 2:**
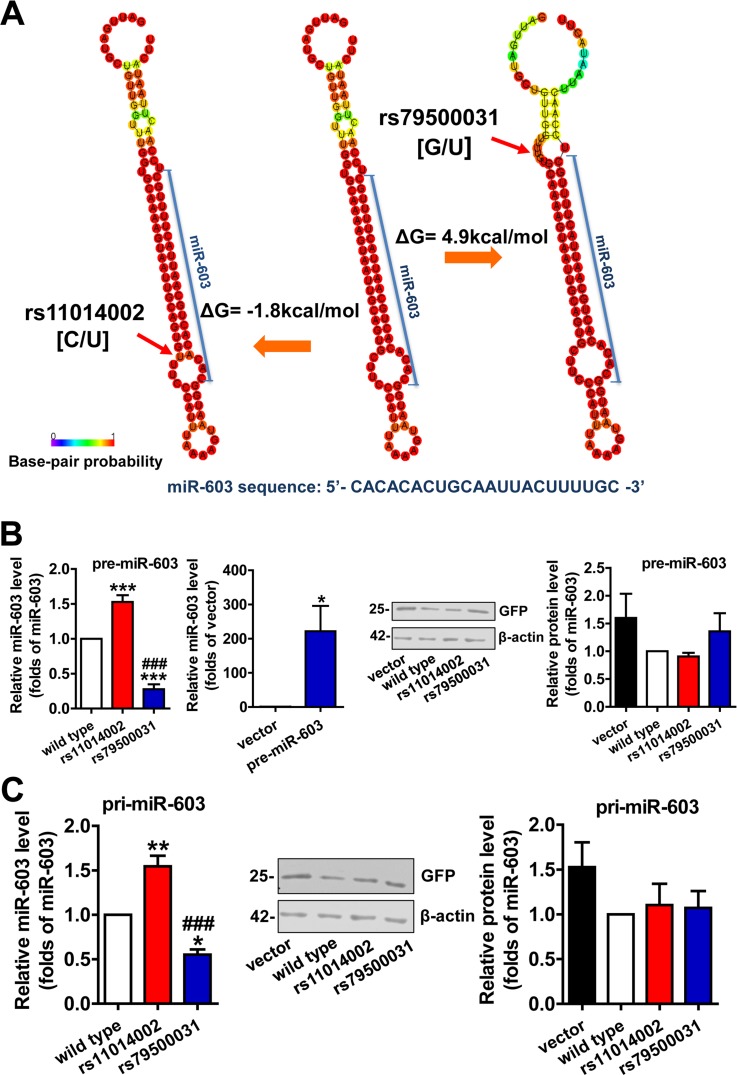
The rs11014002 SNP promotes miR-603 biogenesis (**A**) Predicted secondary structure model and ΔG analysis of miR-603 precursor (pre-miR-603) wild type, pre-miR-603 rs11014002 and pre-miR-603 rs79500031. (**B**) Quantitative RT-PCR analysis of miR-603 expression in the precursor group (left) and immunoblot analysis of GFP expression to confirm transfection efficiency (right). (**C**) Quantitative RT-PCR analysis of miR-603 expression in the primary transcript group (left) and immunoblot analysis of GFP expression to confirm transfection efficiency (middle and right). ***P < 0.001 versus the wild type, *###P* < 0.001 versus the rs11014002 as determined by One-way ANOVA with a Bonferroni's multiple comparison test. *P < 0.05 versus the vector as determined by paired two-tailed Student's t-tests (**B** right).

The enhancement of the stability of the hairpin structure, which is caused by a SNP in a pre-miRNA stem, is believed to increase the production of mature miRNA. Changes in Gibbs free energy (ΔG) can be used to qualify and quantify this process; typically, a negative ΔG is indicative of a more stable structural change and of a higher number of energy changes, to the extent that production is affected [23]. RNAfold analysis of the rs11014002 SNP indicated an alteration that is likely to enhance the stability of the hairpin structure, namely, the substitution of the C allele with a U allele reduces the size of the ring structure. Furthermore, an accompanying ΔG of −1.8 kcal/mol also suggested that the rs11014002 SNP might promote the biogenesis of mature miR-603 (Figure 2A left). Searching miRNASNP, we noted another SNP, rs79500031 in pre-miR-603; in this case, the substitution of the G allele with a U allele produces an asymmetrical ring structure, and the ΔG is 4.9 kcal/mal (Figure 2A right). Similarly, this attenuation of the stability of the hairpin structure appeared to diminish the expression of mature miR-603.

HEK293 cells were transiently transfected with pre-miR-603 wild type, pre-miR-603 rs11014002 and pre-miR-603 rs79500031 plasmids to analyze the effects of these two SNPs on the expression of mature miR-603. The quantitative RT-PCR analysis showed that the rs11014002 SNP increased the expression of mature miR-603 by more than 1.5-fold, whereas the rs79500031 SNP decreased the expression of mature miR-603 drastically, by approximately 70% (Figure 2B left). Compared to the expression of endogenous miR-603 in HEK293 cells, the quantity of mature miR-603 was increased by several hundred fold in the pre-miR-603 transfection group (Figure 2B left). HEK293 cells were also transfected with pri-miR-603 wild type, pri-miR-603 rs11014002, and pri-miR-603 rs79500031 plasmids to confirm the results. Similarly, the rs11014002 SNP increased the turnover of mature miR-603 by 1.5-fold, whereas the rs79500031 SNP decreased the turnover by approximately half (Figure 2C left). GFP expression was used as a control of transfection efficiency, and transfection efficiency did not significantly differ between different groups (Figure 2B right and 2C middle, right). Collectively, these results supported the hypothesis that the rs11014002 SNP in pre-miR-603 can promote the biogenesis of miR-603 and that this SNP may be one of factors that account for differences in the risk of AD.

### miR-603 directly targets LRPAP1 by binding the 3′ untranslated regions (3′UTRs) of LRPAP1 and downregulates LRPAP1 mRNA and protein levels

**Figure 3:**
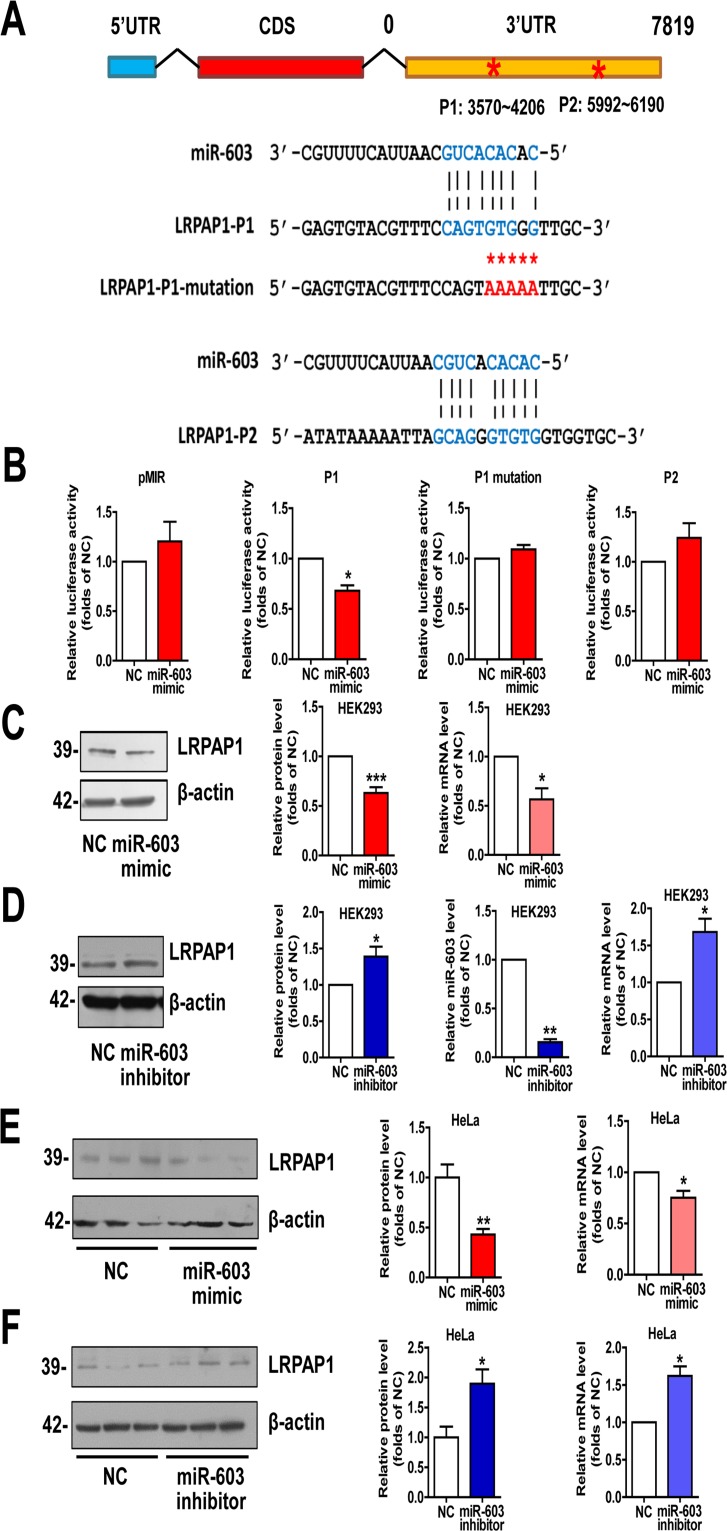
miR-603 directly targets LRPAP1 by binding the 3′UTR of LRPAP1 and downregulates LRPAP1 mRNA and protein levels (**A**) Alignment of potential binding sites for miR-603 in the 3′UTR of LRPAP1 by PITA; scrambled sequences are also included. (**B**) Verification of the miR-603 target sites in the 3′UTR of LRPAP1 by dual-luciferase reporter assay. (**C**) Quantitative RT-PCR analysis of mRNA (right) and immunoblot analysis of protein (left) levels of LRPAP1 after the overexpression of miR-603 in HEK293 cells. (**D**) Quantitative RT-PCR analysis of mRNA (right) and immunoblot analysis of protein (left) levels of LRPAP1 following the inhibition of endogenous miR-603 in HEK293 cells. Quantitative RT-PCR analysis of miR-603 following its inhibition (right) in HEK293 cells. (**E**, **F**) Quantitative RT-PCR analysis of mRNA (right) and immunoblot analysis of protein (left) levels of LRPAP1 following the overexpression of miR-603 (E) and the inhibition of endogenous miR-603 (**F**) in HeLa cells. *P < 0.05, **P < 0.01, ***P < 0.001 versus NC as determine by paired two-tailed Student's *t*-tests for luciferase activity analysis, quantitative RT-PCR analysis (**B**-**F**), and immunoblot analysis (**B**-**D**) and by unpaired two-tailed Student's *t*-tests for immunoblot analysis (**E**, **F**).

We used miRDB algorithm to further explore whether miR-603 targets any AD-related molecules. Among the predicted genes with high scores, we observed that the low-density lipoprotein receptor-related protein associated protein 1 (LRPAP1) is involved in the pathogenesis of AD [24, 25]. The two sites in the 3′UTR of LRPAP1 with the most potential for miR-603 binding were predicted by PITA algorithm and were designated as P1 and P2 (Figure 3A). Dual luciferase assays revealed that the Fluc activity of the reporter that was carrying P1 was markedly diminished in response to miR-603 overexpression. Unlike P1, P2 did not have any effect on Fluc activity, suggesting that P2 is probably not a recognition element of miR-603. We then scrambled the seed region sequences of P1 and found that this scrambled sequence effectively reversed the reduction of Fluc activity (Figure 3B). Additionally, we observed that the LRPAP1 mRNA and protein levels were both reduced when miR-603 was overexpressed (Figure 3C) but were increased when a miR-603 inhibitor was introduced (Figure 3D). We also confirmed these results in HeLa cells (Figure 3E and 3F). Collectively, these results suggested that miR-603 can downregulate the LRPAP1 mRNA and protein levels by directly binding to its 3′UTR.

### miR-603 increases the LRP1 protein levels but not mRNA levels

**Figure 4:**
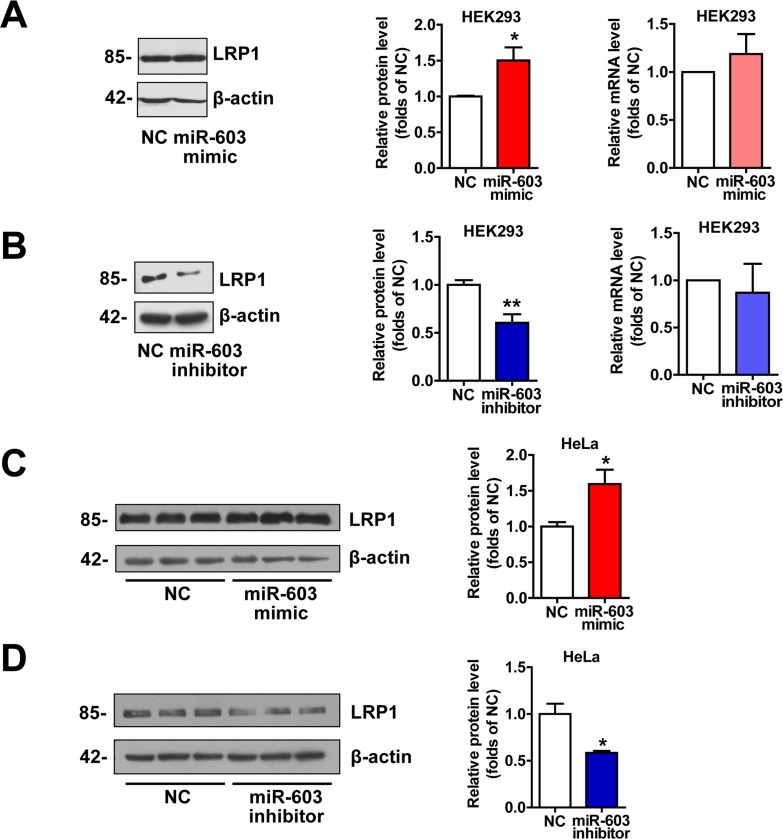
miR-603 increases the LRP1 protein levels (**A**) Quantitative RT-PCR analysis of mRNA (right) and immunoblot analysis of protein (left) levels of LRP1 following the overexpression of miR-603 in HEK293 cells. (**B**) Quantitative RT-PCR analysis of mRNA (right) and immunoblot analysis of protein (left) levels of LRP1 following the inhibition of endogenous miR-603 in HEK293 cells. (**C**, **D**) Immunoblot analysis of protein levels of LRP1 following the overexpression of miR-603 (**C**) and the inhibition of endogenous miR-603 (**D**) in HeLa cells. *P < 0.05, **P < 0.01, ***P < 0.001 versus NC as determined by paired two-tailed Student's *t*-tests for quantitative RT-PCR analysis and by unpaired two-tailed Student's *t*-tests for immunoblot analysis.

LRP1 is an important molecule involved in the pathogenesis of AD by modulating APP processing and Aβ generation [26, 27] and serving as a powerful Aβ-efflux driver of brain through pathways that could be precluded by LRPAP1 [28-30]. Additionally, LRP1 plays a critical role in neurodegeneration prevention through maintaining brain lipid homeostasis and associating synaptic and neuronal integrity [31]. Thus we detected the expression of LRP1. The results indicated that overexpression of miR-603 increased the protein level of low-density lipoprotein receptor-related protein 1 (LRP1) (Figure 4). However, the levels of LRP1 mRNA remained unaltered (Figure 4A and 4B).

### Profiles of differentially expressed genes (DEGs) targeted by miR-603 as determined by Gene Ontology (GO) and pathway analysis

**Figure 5:**
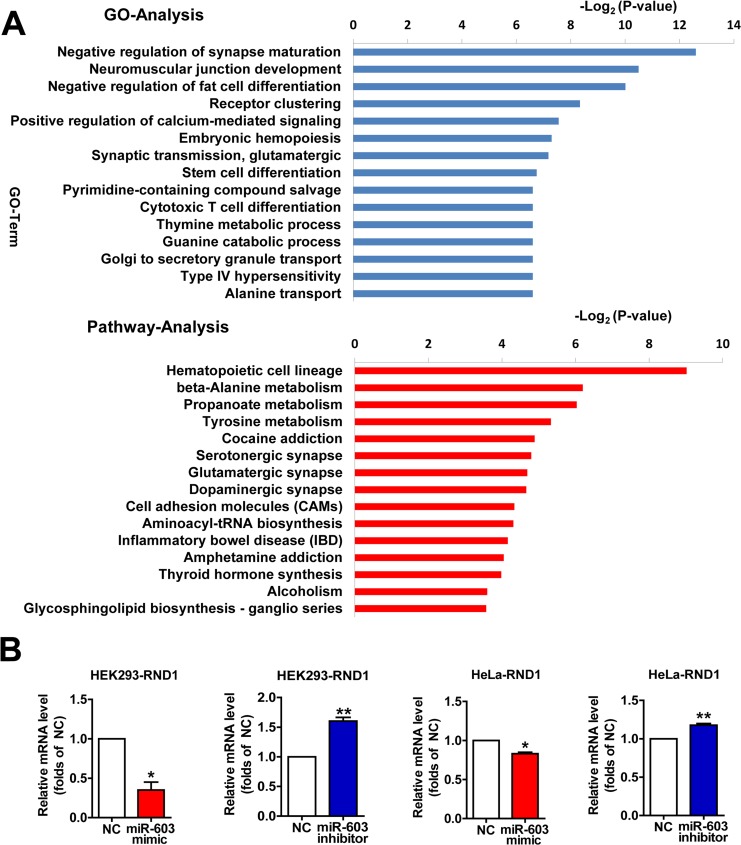
Profiles of differentially expressed genes (DEGs) targeted by miR-603 as determined by Gene Ontology (GO) and pathway analysis (**A**) The fifteen most significantly downregulated biological processes and the fifteen most significantly downregulated pathways in HEK293 cells are shown. Fisher's exact test and χ2 test were used to classify the GO category and to select the significant pathway, and the threshold of significance was identified by *P* <0.05 and FDR < 0.05. (**B**) Quantitative RT-PCR analysis of RND1 mRNA levels following the overexpression of miR-603 and the inhibition of miR-603 in HEK293 cells and HeLa cells. *P < 0.05, **P < 0.01, ***P < 0.001 versus NC as determined by a paired two-tailed Student's *t*-tests for quantitative RT-PCR analysis.

To characterize the functional consequences of gene expression changes induced by miR-603, we performed RNA sequencing on HEK293 cells. When miR-603 was overexpressed, we noted that some significantly downregulated biological processes and pathways were related to functions of the nervous system (Figure 5A). Using quantitative RT-PCR, we verified that the mRNA level of one of these DEGs, Rho family GTPase 1 (RND1), could be downregulated by miR-603, and we confirmed this result in HeLa cells (Figure 5B). RND1 has been implicated in the regulation of neurite out-growth, dendrite development, and axon guidance through its effects on actin dynamics, which are antagonistic to Rho [38]. These results suggest that miR-603 may play roles in the development and functions of the nervous system through its downstream genes and pathways.

### miR-603 exhibits a relatively higher expression in the hippocampi of patients with AD

**Figure 6:**
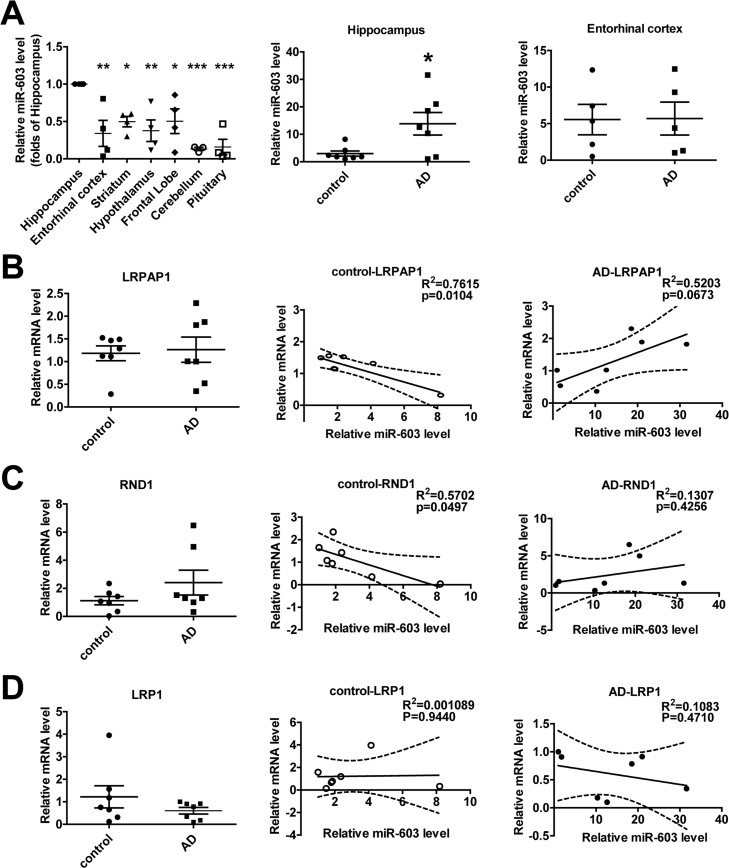
miR-603 exhibits a relatively higher expression in the hippocampi of patients with AD (**A**) Quantitative RT-PCR analysis of miR-603 expression in brain samples (n = 4 for each group, left), *P < 0.05, **P < 0.01, ***P < 0.001 versus the hippocampus as determined by one-way ANOVA with a Dunnett multiple comparison test. Quantitative RT-PCR analysis of miR-603 expression in the hippocampi (middle) and entorhinal cortexes (right) of control subjects and patients with AD (n = 7 for each group); *P < 0.05 as determined by an unpaired two-tailed Student's *t*-test. (**B**) Quantitative RT-PCR analysis of LRPAP1 mRNA levels in the hippocampi of control subjects and patients with AD (n = 7 for each group, left) and linear regression analysis of the correlation between miR-603 expression and LRPAP1 mRNA levels in control groups (middle) and AD groups (right). (**C**) Quantitative RT-PCR analysis of RND1 mRNA levels in the hippocampi of control subjects and patients with AD (n = 7 for each group, left) and linear regression analysis of the correlation between miR-603 expression and RND1 mRNA levels in control groups (middle) and AD groups (right). (**D**) Quantitative RT-PCR analysis of LRP1 mRNA levels in the hippocampi of control subjects and patients with AD (n = 7 for each group, left) and linear regression analysis of the association between miR-603 expression and LRP1 mRNA levels in control groups (middle) and AD groups (right). All the subjects were identified by allocated numbers. The miR-603 expression and LRPAP1, RND1, LRP1 mRNA levels of each subject were standardized by the corresponding values of subject No. 78.

In control group, we found that, compared to other regions of the brain, miR-603 expression is relatively higher in the hippocampus, a region that is affected early and severely in patients with AD (Figure 6A left). Surprisingly, we additionally found that miR-603 levels in the hippocampus, but not in the entorhinal cortex, were significantly higher in the AD group (Figure 6A middle and right).

We monitored the mRNA level of LRPAP1 and the LRPAP1 mRNA level did not differ significantly (Figure 6B left). However, we noted that miR-603 expression was negatively correlated with LRPAP1 mRNA levels in the control group (Figure 6B middle), whereas an opposite correlation appeared to be associated with the AD group (Figure 6B right). We also obtained results for the levels of RND1 mRNA similar to those that were produced when evaluating LRPAP1 (Figure 6C). Specifically, as LRP1 mRNA quantities have been proven to be unaffected by miR-603, we obtained different results for LRP1 compared to those that were produced when evaluating LRPAP1 and RND1 (Figure 6D).

### miR-603 downregulates the expression of E2F transcription factor 1 (E2F1) and prevents HeLa cells from undergoing H_2_O_2_ –induced apoptosis

**Figure 7:**
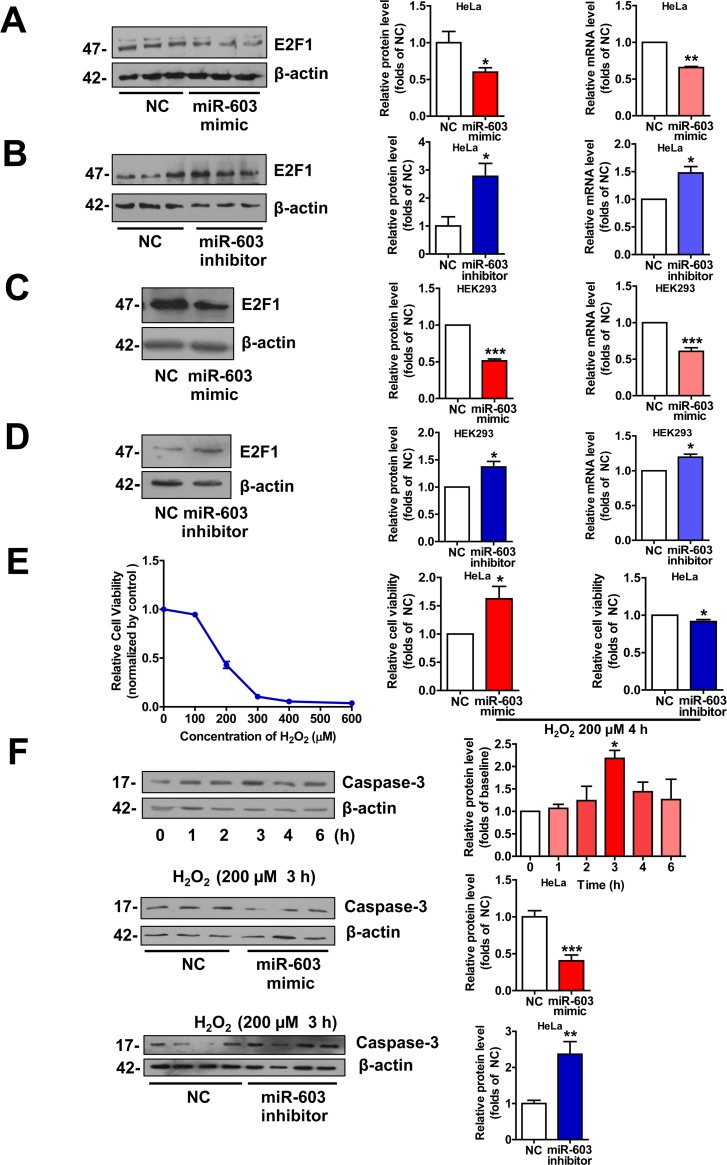
miR-603 downregulates E2F1 expression and prevents HeLa cells from undergoing H_2_O_2_–induced apoptosis (**A**) Quantitative RT-PCR analysis of mRNA (right) and immunoblot analysis of protein (left) levels of E2F1 following the overexpression of miR-603 in HeLa cells. (**B**) Quantitative RT-PCR analysis of mRNA (right) and immunoblot analysis of protein (left) levels of E2F1 following the inhibition of endogenous miR-603 in HeLa cells. (**C**, **D**) Quantitative RT-PCR analysis of mRNA (right) and immunoblot analysis of protein (left) levels of E2F1 following the overexpression of miR-603 (C) and the inhibition of endogenous miR-603 (**D**) in HEK293 cells. *P < 0.05, **P < 0.01, ***P < 0.001 versus NC as determined by paired two-tailed Student's *t*-tests for quantitative RT-PCR analysis and immunoblot analysis. (**E**) Analysis of cell viability after H_2_O_2_-induced oxidative stress following the overexpression of miR-603 and the inhibition of miR-603 in HeLa cells (middle and right; left is the concentration-dependent effect test). (**F**) Immunoblot analysis of cleaved Caspase 3 expression after H_2_O_2_-induced oxidative stress following the overexpression of miR-603 (above) and the inhibition of miR-603 (below) in HeLa cells. *P < 0.05 versus the 0 h as determined by One-way ANOVA with a Dunnett multiple comparison test (F, Top). *P < 0.05, **P < 0.01, ***P < 0.001 versus NC as determined by paired two-tailed Student's *t*-tests for cell viability analysis and by an unpaired two-tailed Student's *t*-test for immunoblot analysis.

E2F1 is a key component in the neuronal apoptotic route, and its expression is sufficient to induce death in cortical neurons in the absence of apoptotic stimuli. In addition, the neuronal apoptosis that is induced by Aβ is mediated by E2F1, and Aβ can promote E2F1 expression [9, 39, 40]. As a target of miR-603 [41], we hypothesized that miR-603 may protect neurons from undergoing apoptosis in patients with AD by downregulating E2F1. We first confirmed that miR-603 diminished the E2F1 mRNA and protein expression levels in HeLa cells (Figure 7A and 7B) and in HEK293 cells (Figure 7C and 7D). Then, we conducted cell viability analysis following the application of 200 μM H_2_O_2_ to mimic Aβ-dependent apoptosis in HeLa cells; this concentration was determined to be sufficient for our needs (Figure 7E left). We found that miR-603 overexpression increased cell viability, whereas miR-603 inhibition decreased cell viability (Figure 7E middle and right). miR-603 overexpression consistently decreased the level of cleaved caspase 3, an apoptosis indicator, whereas miR-603 inhibition increased it (Figure 7F). The above results supported the possible anti-apoptotic role of miR-603 via its downregulation of E2F1 in the H_2_O_2_-induced apoptosis system.

## DISCUSSION

**Figure 8:**
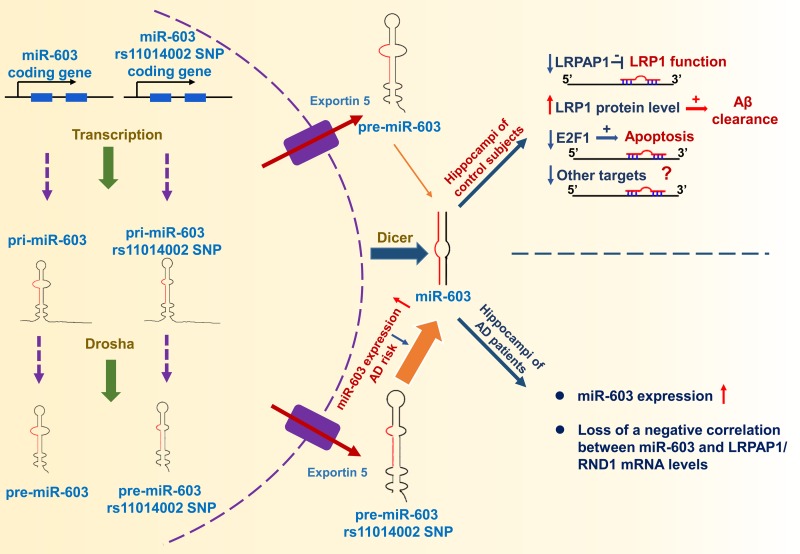
Working model The rs11014002 SNP in pre-miR-603 increases the expression of miR-603, which may account for a reduced AD risk. miR-603, which targets LRPAP1 and increases the LRP1 protein levels, may promote Aβ clearance in the brain. miR-603 can prevent cells from undergoing apoptosis possibly by downregulating E2F1. In the hippocampi of patients with AD, miR-603 loses its regulatory role and manifests a compensatory increase.

Through ADNI data analysis, we found that the rs11014002 SNP carriers exhibit reduced risk of AD and the rs11014002 SNP may serve as a protective factor against AD. Additionally, the rs11014002 SNP could promote miR-603 biogenesis. Moreover, we demonstrated that miR-603 directly targets LRPAP1 and upregulates LRP1 protein levels. Intriguingly, we found that miR-603 exhibits a relatively higher expression and there is a loss of a negative correlation between miR-603 and LRPAP1/RND1 mRNA levels in the hippocampi of patients with AD. Furthermore, miR-603 can prevent HeLa cells from undergoing H_2_O_2_-induced apoptosis possibly through targeting E2F1. Based on the above results, we proposed a working model that depicts the functional significance of miR-603 regarding AD pathogenesis and risk (Figure 8).

According to previous studies, LRPAP1 not only inhibits the binding of almost all known ligands of LRP1[32-34], but also controls the expression of LRP1[35] by acting as a molecular chaperone of LRP1[36]. LRPAP1 knockout leads to a significant decrease in LRP1 and ultimately results in decreased Aβ clearance due to inhibition of LRP1 maturation [37]. However, in our study, when miR-603 downregulated LRPAP1, the LRP1 protein levels significantly increased, in contrast to the anticipated decrease in these levels. Clearly, in its capacity as a master regulator of transcriptional networks, miR-603 likely employs various pathways that can balance and even surpass the influence of LRPAP1 on LRP1. The specific mechanisms behind these findings remain to be explored. As miR-603 increased the expression of LRP1 and potentially enhanced the function of LRP1 through downregulating LRPAP1, we hypothesized that miR-603 may have a protective role with respect to the pathogenesis of AD. This role would also help to explain how subjects that carry the rs11014002 SNP have a reduced AD risk because a relatively higher level of miR-603 leads to a higher level of LRP1, thereby affording a greater protective effect against AD.

The highly and widely expressed LRP1 functions to maintain brain homeostasis and to control Aβ metabolism in a variety of cell types, including neurons, vascular cells and glial cells [28]. Decreases in LRP1 levels have been observed in the middle frontal cortexes of patients with AD; higher LRP1 levels significantly correlate with later ages of AD onset, whereas age and LRP1 expression appear to be inversely correlated in unaffected individuals [42]. Taken together, the positive effect of miR-603 on the expression, and potentially on the function, of LRP1 supports its protective role against AD.

E2F1 is a key neuronal apoptosis-related molecule that is involved in AD pathogenesis [43]. Additionally, E2F1 coordinates a large group of genes that are involved in regulating the G1 to S phase transition [44]. Endogenous G1 cell cycle regulators are known to play important roles in toxicity-induced neuronal apoptosis in patients with AD [45]. Therefore, miR-603 may also protect neurons from apoptosis via downregulation of the cell cycle. GO analysis of our RNA sequencing results also suggested that miR-603 has a tendency to downregulate the G1 to S transition of the mitotic cell cycle. However, these hypotheses must be verified in the future.

The alteration of miRNAs expression has been associated with several pathological processes. To be specific, the dysregulation of miRNAs in brains of AD patients have been reported by an increasing number of studies. Some of these miRNAs have been consistently identified as AD-specific miRNAs and their targets also seem implicated in pathophysiological processes underlying AD. These miRNAs seem to fall into three situations:

1. miRNAs (e.g. miR-9[46], miR-107[47, 48], miR-29[8]) that downregulate/have a negative correlation with “pro-AD” genes (e.g. FGFR1, NFκB, SIRT1, BACE1, CDK5, ADAM10)/are negatively correlated with amount of Aβ plaque and NFTs, exhibit downregulated in the brain and/or peripheral circulation of AD patients.
2. miRNAs (e.g. miR-146a[49], miR-34[50], miR-181[51]) that downregulate protective genes against AD (e.g., complement factor H)/upregulate “pro-AD” genes (e.g. p53, SIRT1)/are correlated with amount of Aβ plaque and NFTs, exhibit upregulated in the brain and/or peripheral circulation of AD patients.
3. miRNAs (e.g. miR-106a, miR-106b[52]) that play multiple roles in the pathogenesis of AD, exhibit downregulated/upregulated in the brain and/or peripheral circulation of AD patients.

Nevertheless, whether changes in miRNA expression drive neurodegeneration or whether changes in miRNA expression simply arises during the course of the disease remains controversial [8, 53]. Addition of Aβ peptides to primary neuronal cell cultures/primary human astrocytes cultures has been shown to downregulate miR-9/miR-181[51]. Based on this, we could not rule out the possibility that the dysregulation or aberrant expression of miRNAs may be secondary to the AD pathology. Concerning our results, the loss of a negative correlation between miR-603 and LRPAP1 and RND1 mRNA levels may suggest that miR-603 is dysregulated with respect to its target genes in response to AD pathology. Thus, the significantly higher expression of miR-603 in the hippocampi of patients with AD may result from a compensatory feedback mechanism that compensates for the loss of the regulatory role of miR-603. However, another situation could be that AD pathology leads to the aberrant expression of miR-603 directly. Although several discrepancies exist and further validation is required, these observations open the door to potential novel diagnostic and therapeutic tools for patients with AD.

Since miR-603 is primate-specific, the existence of miR-603 and its recognition sites in the 3′UTR of the target genes are not conserved in non-primate animals. So we can't use the frequently-used primary culture of neurons of mice or rats. HEK293 cells[55], HeLa cells[56-59], HEK293 cells overexpressing the amyloid precursor protein (APP) with the Swedish mutation[60] or Tau[61] are widely used in mechanism studies of AD pathogenesis. In these studies, results obtained from these cell lines have also been confirmed in primary culture of neurons. Moreover, the transfection efficiency of HEK293 cells and HeLa cells are higher than some neuronal cell lines such as SHSY5Y cell lines. Since we performed lots of overexpression and inhibition experiments, HEK293 cells and HeLa cells are better tools to insure stable and relatively higher transfection efficiency. Given that miR-603 is a primate-specific miRNA, we do not currently have appropriate animal models to verify these in vitro results. Further studies could be performed on humanized animal models.

SNPs are associated with an individual's risk of acquiring various illnesses and are considered genetic biomarkers for disease assessment. SNPs in AD-associated genes such as *APOE4* [54] and Calcium Homeostasis Modulator 1 (*CALHM1*) [55] have been verified to account for AD risk. Several SNPs have been found in miRNAs (miRSNPs) and are regarded as factors that can regulate the expression or function of miRNA [56]. Although select miRSNPs have recently been found to be involved in nervous system diseases [57], their potential roles in neurodegenerative diseases have not been clarified. This study is the first to report that the rs11014002 SNP in pre-miR-603 is associated with a reduced risk of AD, suggesting that this SNP may serve as a potential genetic biomarker for AD risk assessment.

Therefore, based on a large population data analysis and experimental mechanism studies, our work might have general implications for better understanding the roles of miRNAs and miRSNPs in AD pathogenesis. Importantly, we uncovered a novel miRSNP that may serve as a potential genetic biomarker for AD risk assessment.

## METHODS

### ADNI data

The Alzheimer's Disease Neuroimaging Initiative (ADNI) (http://www.loni.ucla.edu\ADNI) unites researchers with study data as they work to define the progression of Alzheimer's disease. ADNI researchers collect, validate and utilize data such as MRI and PET images, genetics, cognitive tests, CSF and blood biomarkers as predictors for the disease. Data from the North American ADNI's study participants, including Alzheimer's disease patients (AD), mild cognitive impairment subjects (MCI) and cognitively normal elderly controls (NC) are available from this site. We obtained demographic, genetic and magnetic resonance imaging (MRI) data of 365 NC, 772 MCI and 301 AD from the ADNI database. Genotype information was re-trieved from the genetic data of each individual (Table 2).

### Pre-miR-603 secondary structure prediction

An optimal secondary structure of pre-miR-603 with a minimum free energy was predicted by the RNAfold web server (http://rna.tbi.univie.ac.at/cgi-bin/RNAfold.cgi).

Additional information regarding SNPs of miR-603 was provided by the miRNASNP web server (http://www.bioguo.org/miRNASNP/).

### miR-603 target gene prediction

miR-603 target gene prediction was accomplished using the miRDB web server (http://www.mirdb.org/miRDB/) and the PITA web server (http://genie.weizmann.ac.il/pubs/mir07/mir07_prediction.html).

### Cell lines

Human HEK293 cells and HeLa cells were provided by the Cell Resource Center, Institute of Basic Medical Sciences Chinese Academy of Medical Sciences & School of Basic Medicine Peking Union Medical College, Beijing, China.

### Cell cultures and transfections

Human HEK293 cells and HeLa cells were cultured in 35 mm dishes in a humidified atmosphere of 95% air and 5% CO_2_ at 37°C. The culture media was DMEM (Gibco, Carlsbad, CA, USA) supplemented with 10% FBS (HyClone, Logan, UT, USA). Cells were transfected with either a 20 nM miR-603 mimic or a miR-603 inhibitor along with the corresponding negative control (NC) (GenePharma, Shanghai, China) using Lipofectamine 2000 (Invitrogen, Carlsbad, CA, USA). At 48 h post-transfection, the cells were processed for further analysis. The sequences of NC are strictly designed corresponding to the sequences of miR-603 mimic and miR-603 inhibitor:

miR-603: CACACACUGCAAUUACUUUUGC (miRBase Accession number: MI0003616)
miR-603 mimic: CACACACUGCAAUUACUUUUGC AAAAGUAAUUGCAGUGUGUGUU
NC of miR-603 mimic: sense 5′-UUCUCCGAACGU GUCACGUTT-3′
antisense 5′-ACGUGACACGUUCGGAGAATT-3′
miR-603 inhibitor: GCAAAAGUAAUUGCAGUGU GUG
NC of miR-603 inhibitor: 5′-CAGUACUUUUGUGU AGUACAA-3′

### Plasmids

We employed expression plasmids that contained 97-bp long pre-miR-603 coding regions and 408-bp long miR-603 primary transcript (pri-miR-603) coding regions, both of which were under the control of the CMV promoter (GenePharma, Shanghai, China). Moreover, we mutated the sites that corresponded to rs11014002 SNP and rs79500031 SNP. The sequences of these plasmids were confirmed by direct sequencing.

### RNA extraction and quantitative reverse transcription-polymerase chain reaction (qRT-PCR) analysis

After 48 h, total RNA was extracted using TRIzol reagent (Invitrogen, Carlsbad, CA, USA). RNA concentration and purity was measured using NanoDrop 2000c spectrophotometer (Thermo Scientific, Waltham, MA, USA).

Quantitative RT-PCR was performed using an ABI 7500 instrument (Applied Biosystems, Foster City, CA, USA). Every sample in each of the groups was measured in triplicate, and the experiment was repeated at least three times.

TaqMan miRNA reverse transcription reagents (4366596; Applied Biosystems, Foster City, CA, USA) and miR 5× RT primers (hsa-miR-603/RT457, U6/RT515; Applied Biosystems, Foster City, CA, USA) were used for reverse transcription. Ten nanograms of RNA were used as the initial input. RT protocol: 30 minutes at 16°C; 30 minutes at 42°C; 5 minutes at 85°C and hold at 4°C. To investigate the expression of specific miRNA, we used TaqMan Universal PCR Master Mix (4324018; Applied Biosystems, Foster City, CA, USA) with miRNA-specific TaqMan MGB probes (hsa-miR-603/TM457, U6/TM515; Applied Biosystems, Foster City, CA, USA). Real-time PCR protocol: hold 10 minutes at 95°C; 40 cycles (denature 15 sec at 95°C; anneal/extend 60 sec at 60°C).

For mRNA expression analysis, single-stranded cDNA was synthesized using Reverse Transcription MasterMix (catalog # G486; Applied Biological Materials, Richmond, Canada). RT protocol: 10 minutes at 25°C; 15 minutes (for qPCR) or 50 minutes (for PCR) at 42°C; 5 minutes at 85°C and hold at 4°C. Gene expression levels were detected using SYBR Green (catalog # QPK-201; Toyobo, Japan). Real-time PCR protocol: 60 sec at 95°C; 40 cycles (15 sec at 95°C; 15 sec at 60°C; 45 sec at 72°C); melting curve analysis. miRNA data was normalized by the expression of U6; mRNA data was normalized by the expression of GAPDH.

### Reporter plasmid construction and dual luciferase assays

The mammalian expression vector pMIR-REPORTTM luciferase expression plasmid (Promega, Madison, WI, USA) expressing Fluc and pRL-TK luciferase expression plasmids (Promega, Madison, WI, USA) expressing Rluc are used. P1 (cagtgtggg) residing in the 3′UTR of LRPAP1 (NM_ 002337.3) between nucleotides 3570 and 4206, and P2 (gcagggtgtg) residing in the 3′UTR of LRPAP1 (NM_ 002337.3) between nucleotides 5992 and 6190, are predicted to be with the most potential for miR-603 binding. For P1, we cloned 199 nucleotide sequences containing the predicted binding site of the miR-603 seed region (cagtgtggg) into the 3′UTR of the Fluc gene at HindIII and SacI restriction sites in the reporter plasmid; for P2, we cloned 640 nucleotide sequences containing the predicted binding site of the miR-603 seed region (gcagggtgtg) into the 3′UTR of the Fluc gene at HindIII and SacI restriction sites in the reporter plasmid. Mutated P1 (cagt**aaaaa**) contains sequence modifications that are predicted to affect the “seed region” that is targeted by miR-603. Construct sequences were verified by sequencing.

For the luciferase reporter assay, HEK293 cells were plated at a density of 4×10^5^ per well in a 24-well plate one day before transfection. In total, 100 ng of pMIR reporter plasmid and 50 ng of pRL-TK control plasmid along with 20 nM miR-603 mimic or control were transfected using Lipofectamine 2000 (Invitrogen, Carlsbad, CA, USA). Luciferase reporter activity analysis was performed 24 h after the transfection using a Dual-Luciferase Assay System (Promega, Madison, WI, USA) on a Centro XS^3^ LB 960 (Berthhold Technologies, Bad Wildbad, Germany). To control for transfection efficiency, relative luciferase activity was alculated by normalizing the firefly luciferase (Fluc) to the renilla luciferase (Rluc) readouts. Each experimental condition was performed in triplicate within individual experiments; the results that are shown represent three independent experiments.

### RNA sequencing

To characterize the functional consequences of gene expression changes induced by miR-603, we overexpressed negative control (NC) and miR-603 mimic in HEK293 cells and performed RNA sequencing. After 48 h, total RNA was extracted using TRIzol reagent (Invitrogen, Carlsbad, CA, USA). The quality of RNA was assessed using an Agilent 2200 (Agilent Technologies, San Diego, CA, USA). Using high-throughput Life technologies Ion Proton Sequencer, the transcript with poly(A)-containing RNA of Human were analyzed. The Differentially expressed genes (DEGs) were achieved by EB-Sequencing algorithms and annotated by NCBI Database. We selected the DEGs according to the false discovery rate (FDR) threshold set at p < 0.05 and FDR <0.05. And the fold changes of two groups are more than 2. Gene Ontology (GO) analysis was applied to analyze the primary functions of the DEGs according to the GO. Similarly, pathway analysis was used to determine the significant pathways of the DEGs according to the KEGG database. Generally, Fisher's exact test followed by Benjamini–Hochberg (BH) multiple testing correction was calculated to select the GO category and the significant pathway. The significant GO terms and significant pathways were defined as P < 0.05. RNA-sequencing data sets were deposited in the NCBI Gene Expression Omnibus database (GEO GSE68987).

### Western blot analysis

Cell cultures were washed twice with ice-cold PBS and lysed in ice-cold lysis buffer (50 mM Tris-HCl, pH7.4, 150 mM NaCl, 1.5 mM MgCl_2_, 10% glycerol, 1% Triton X-100, 5 mM EGTA, 1 ng/ml leupeptin, 1 mM PMSF, 1 mM Na_3_VO_4_, 10 mM NaF, and proteinase inhibitor mixture). The lysates were centrifuged at 12,000×*g* for 5 min to obtain the total protein extract from the supernatant. The protein concentration was measured using a BCA assay kit (Pierce Biotechnology, Rockford, IL, USA). Equal amounts of samples (50 ng) were denatured and subjected to 10% SDS-PAGE. After separation, proteins were transferred to nitrocellulose membranes (Bio-Rad, Hercules, CA, USA). The membranes were blocked with 5% nonfat milk in TBST for 1 h at room temperature and incubated with primary antibody overnight at 4°C. After the membranes were washed three times with TBST, they were incubated with horseradish peroxidase (HRP)-conjugated secondary antibody for 1 h at room temperature, washed again, and finally developed with ECL solution (ThermoFisher Scientific, Waltham, MA, USA). The immunoreactive bands were scanned and quantitatively analyzed by densitometry with Quantity One (Bio-Rad, Hercules, CA, USA). The Western blot data is normalized on the signal of β-actin.

### Antibodies

The commercial antibodies that were used included anti-E2F1 (catalog # AP7593b; Abgent, San Diego, CA, USA), anti-LRPAP1 (catalog # AP8529b; Abgent, San Diego, CA, USA), anti-LRP1 (catalog # AJ1448a; Abgent, San Diego, CA, USA), anti-Caspase-3 (catalog # YT0656; ImmunoWay, TX, USA), anti-GFP (catalog # AE012; ABclonal, Cambridge, MA, USA), and anti-actin (Sigma-Aldrich, St. Louis, MO, USA).

Dilution of the primary antibodies: anti-E2F1 (1:500), anti-LRPAP1 (1:500), anti-LRP1 (1:10000), anti-Caspase-3 (1:1000), anti-GFP (1:1000), and anti-actin (1:5000).

### Human sample analysis

Brain tissues were obtained from the Human Brain Bank, Neuroscience Center, Chinese Academy of Medical Sciences & Peking Union Medical College, Beijing, China, under an Institutional Review Board protocol. Subjects who offered human brain tissues were identified by numbers, not by names. Studies using human brain tissues were ethically approved and supervised by the Human Brain Bank, Neuroscience Center, Chinese Academy of Medical Sciences & Peking Union Medical College. Cases were separated into two groups: a control group and an AD group (n=7, respectively, Table 3). Premortem clinical evaluations were exhaustive, and control samples were obtained from the brains of patients with no AD-type clinical manifestation and those with no AD history. Clinical diagnoses were established at a consensus conference with neuropsychologists, neurologists, and neuropathologists from the Human Brain Bank, Neuroscience Center, Chinese Academy of Medical Sciences & Peking Union Medical College.

**Table 3:** Cases included in our study

Control ID	Gender	Age	Medical history	PMI (h)	
PTB055	F	84	coronary heart disease	5	
PTB058	F	90	respiratory failure	18	
PTB065	M	94	respiratory failure	70	
PTB067	M	90	lung cancer	11	
PTB069	M	92	organ failure	16.5	
PTB013	F	76	lung cancer	8	
PTB023	F	81	lung disease	12	
Average		86.7143		20.0714	
AD ID	Gender	Age	Medical history	PMI (h)	Stage of AD
PTB050	F	80	cerebral thrombosis, pulmonary embolism	4.5	AD
PTB062	F	98	respiratory failure	69	AD
PTB078	F	86	organ failure	6.33	mild AD
PTB083	F	100	hypertension	3	manic AD
PTB051	M	89	chronic bronchitis, arrhythmia	5	moderate AD
PTB029	F	73		17	pre-AD stage
PTB048	F	82	chronic emphysema	4	mild AD
Average		86.8571		15.5471	

PMI: Postmortem interval.

RNA was isolated from brain tissues (0.5 g) that had been snap-frozen in liquid nitrogen and was then transferred to a −80°C freezer. Tissue was homogenized for 5 min on ice using a glass and Teflon hand homogenizer followed by additional disruption with a Microson XL-2000 hand sonicator (Fisher Scientific, Chelmsford, MA, USA) (three times for 10 s each on ice). The samples were then centrifuged at 16,000×*g* for 15 min at 4°C. The supernatant was used for RNA isolation. TRIzol reagent (Invitrogen, Carlsbad, CA, USA) was used according to the manufacturer's instructions, except for an additional overnight −20°C isopropanol precipitation. RNA concentration and purity were measured via spectrophotometry (Bio-Rad, Hercules, CA, USA) using a NanoDrop 2000 (Thermo Scientific, Waltham, MA, USA); RNA integrity was measured by using electrophoresis to monitor the ratio between 28s and 18s. Degraded samples were discarded.

All assays were performed by operators blinded to the clinical features of the samples.

### Cell viability analysis using a CCK8 kit

HeLa cells were plated in 96-well plates (1.5 × 10^4^ cells/well) one day before transfection and then transfected with either a miR-603 mimic (20nM) or inhibitor (20nM) along with the corresponding negative control (NC) (20nM) (GenePharma, Shanghai, China). After 48 h, the culture medium in each well was replaced by DMEM containing 200 μM H_2_O_2_ without FBS for 4 h. Following this, the culture media in each well was replaced by 10 μl of CCK8 (Solarbio, Beijing, China) and 90 μl of DMEM. After incubation for 2 h at 37°C, the absorbance of each well was detected using a microplate reader at a wavelength of 450 nm. The cell viability of wells with no cells placed and with CCK8 was used as an empty control “A_(0)_”; wells with cells placed, without H_2_O_2_ and with CCK8 was used as “A_(1)_”; wells with cells placed, with H_2_O_2_ in different concentration and with CCK8 was used as “A_(e)_”. The calculating formula is as follow: Relative cell viability (%) = (A_(e)_- A_(0)_)/( A_(1)_- A_(0)_) ×100%.

### Randomization

Cells were randomly assigned to experimental groups and to the processing order, and human brain samples were randomly assigned to the processing order.

Statistical analyses. The data in the figures represent the mean ± SEM. Comparisons between two groups were performed using Student's unpaired or paired 2-tailed t-tests depending on the manner in which the experiments were conducted. Comparisons among three or more groups were performed using one-way ANOVA analyses followed by Bonferroni's multiple comparison test or a Dunnett multiple comparison test depending on the manner in which the experiments were conducted. Linear regression was used to examine the relationships between miR-603 expression and LRPAP1, RND1, LRP1 mRNA levels in control and AD groups. The data marked with asterisks in the figures significantly differ from the controls as follows: *P < 0.05, **P < 0.01, ***P <0.001.

## SUPPLEMENTARY INFORMATION


