# Supplementary material for: Primate-specific miR-603 is implicated in the risk and pathogenesis of Alzheimer's disease
Source: Aging (Albany NY). 2016 Feb 8;8(2):272–90. doi: 10.18632/aging.100887 (PMC4789582; doi:10.18632/aging.100887)
Supplement: Supplementary file 1 [file aging-08-272-s001.pdf]

Supplementary information

1 LD analysis

(1) The rs11014002 SNP is locating at 24275724 on the chromosome 10. We performed the LD analysis of all the SNPs 10kb upstream and downstream of 24275724 among the American population (sample size: n=503) from the Ensembl project (http://asia.ensembl.org/index.html). The results are shown as follows:

D’:

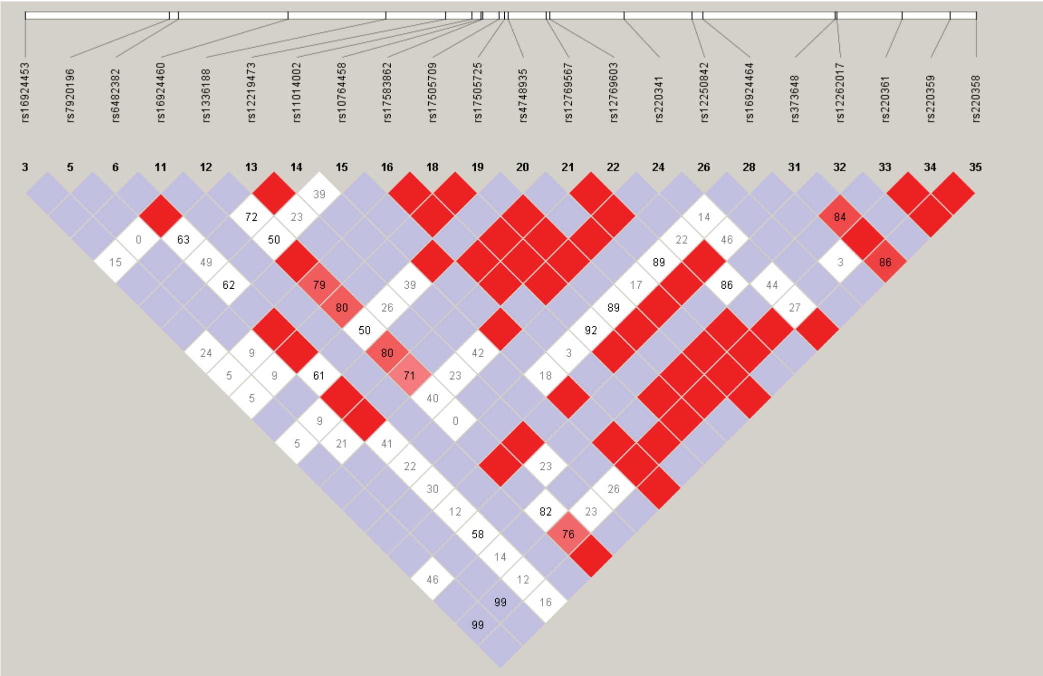

r^2:

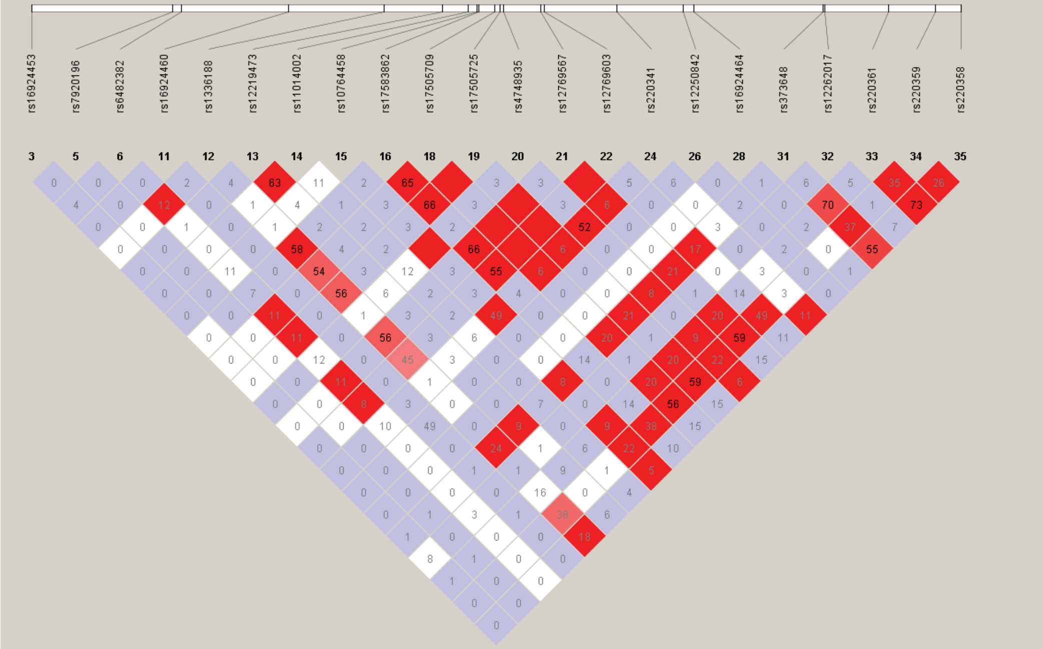

(2) SNPs with possible LD association and their calculated values are listed as follows:

D'

SNP	values
rs7919749	1
rs10741049	1
rs1336187	1
rs11014001	1
rs12219473	1

r^2

SNP	values
rs7919749	0.623
rs10741049	0.062
rs1336187	0.065
rs11014001	0.054
rs12219473	1

(3) However, these SNPs haven't been reported to be involved in AD. The most possible LD association appears to be between rs12219473 and rs11014002.

2 RNA Sequencing

Whole transcriptome libraries preparation and deep sequencing

The sequencing library of each RNA sample was prepared by using Ion Total RNA-Seq Kit v2 according to the protocol provided by manufacturer (Life technologies, USA). Briefly, poly(A)-containing mRNA was purified from 5 ug total RNA with Dynabeads (Life technologies, USA). The mRNA was fragmented using RNaseIII and purified. The fragmented RNA was hybrized and ligated with Ion adaptor. The RNA fragments were reversetranscribed and amplified to double-stranded cDNA. Then, the amplified cDNA was purified by magnetic bead based method, and the molar concentration was determined for each cDNA library. Emulsion PCR was performed using template of cDNA library. The TemplatePositive Ion PI™ Ion Sphere™ Particles were enriched and loaded on the Ion PI™ chip for sequencing.

Raw Data Treatment

Using high-throughput Life technologies Ion Proton Sequencer, the transcript with poly(A)-containing RNA of Human were analyzed. Reads sequenced were filtered and mapped to Human genome (download from NCBI) using Mapsplice software. The mapped reads was counted to achieve the expression of each gene based on the gene annotation information from NCBI database.

Data Filtering

In order to achieve the best quality of the RNA Sequencing, Novelbio applied the reads filtration to filter the reads with lower quality and short sequence under following criteria: read length > 50; over 30% base quality >13. Other quality control result was showed in supplementary datasheet and could be achieved in file “1.FastQC” including the quality score indicating the reads in visualized

ways and Sequence GC Content indicating no other species pollution in experiment.

Mapping Statistics

Based on the clean reads after filtering, Novelbio applied the RNA-seq mapping using Mapsplice software to the Human genome for further study. Mapping statistics was shown in Table as follow, from which we could mention the mapping rates about 85% indicating the well-performance of the sequencing experiment. Furthermore, the unique mapping rate is more than 81%， which could lead to the best quality of the gene expression.

Statistics Term	Result (miR-603)	Result (NC)
allReads	25144459	24871786
UnMapped	2741967	3724949
MappedReads	22402492	21146837
MappingRate	0.891	0.85
UniqueMapping	21539217	20155452
UniqueMappingRate	0.857	0.81
repeatMapping	863275	991385
junctionAllMappedReads	5837949	4770294
junctionUniqueMapping	5828232	4759745
insertSize	72006382	71601751

Preliminary Analysis

The differentially expressed genes were achieved by EB-Seq algorithms and annotated by NCBI Database and by blasting to Arabidopsis protein sequence of the transcripts. Based on the differentially expressed genes, we applied the Gene Ontology (GO) Analysis and Pathway Analysis

to discover the function and pathway enriched among the differentially expressed genes.

Identification of differentially expressed genes

The EB-Seq algorithm was applied to filter differentially expressed genes for the miR-603 and NC groups. After the significance analysis and FDR (false discovery rate) analysis [Benjamini Y, et al. Controlling the false discovery rate in behavior genetics research. Behav Brain Res. 2001 Nov 1;125(1-2):279-84.], we selected the differentially expressed genes according to the FDR threshold set at $p < 0.05$ and FDR <0.05. And the fold changes of any two groups are more than 2.

GO analysis

Gene Ontology (GO) terms were assigned to each differ-gene. GO terms are dynamically-structured control vocabulary that can be applied to describe functions of genes and by which genes can be classified into three major categories, namely Biological Process, Molecular Function, and Cellular Component, and their sub-categories.

Differentially expressed genes were determined from statistical outcomes by testing for association with biological process gene ontology (GO) terms (Gene Ontology Consortium 2006). Fisher's exact test was used to classify the GO category, and the false discovery rate (FDR) was calculated to correct the P value; the smaller the FDR, the smaller the error in judging the P value (Dupuy et al. 2007). Enrichment of GO members among differentially expressed probe sets was found using the one-tailed Fisher's exact test for 2×2 contingency tables (Dunnick et al. 2012), and it provides a measure of the significance of the function that as the enrichment increases, the corresponding function is more specific, which helps us to find those GOs with more concrete function description in the experiment.

Pathway analysis

Similarly, pathway analysis was used to find out the significant pathway of the differential genes according to KEGG database. Still, Fisher's exact test followed by Benjamini–Hochberg (BH) multiple testing correction was calculated to select the significant pathway, and the threshold of significance was defined by P-value and FDR. The significant pathway was identified by P value <0.05 and FDR < 0.05.

3 Genes with high scores and predicted to be regulated by miR-603 in miRDB

Target Detail	Target Rank	Target Score	miRNA Name	Gene Symbol	Gene Description
Details	1	100	hsa-miR-603	MACC1	metastasis associated in colon cancer 1
Details	2	100	hsa-miR-603	C16orf53	chromosome 16 open reading frame 53
Details	3	100	hsa-miR-603	UBN2	ubinuclein 2
Details	4	100	hsa-miR-603	ITGAM	integrin, alpha M (complement component 3 receptor 3 subunit)
Details	5	100	hsa-miR-603	SLITRK4	SLIT and NTRK-like family, member 4
Details	6	100	hsa-miR-603	SLC6A19	solute carrier family 6 (neutral amino acid transporter), member 19
Details	7	99	hsa-miR-603	LRPAP1	low density lipoprotein receptor-related protein associated protein 1
Details	8	98	hsa-miR-603		syntaxin 1B
Details	9	97	hsa-miR-603	SBNO1	strawberry notch homolog 1 (Drosophila)
Details	10	97	hsa-miR-603	USP36	ubiquitin specific peptidase 36
Details	11	97	hsa-miR-603	SERBP1	SERPINE1 mRNA binding protein 1
Details	12	97	hsa-miR-603	C1orf216	chromosome 1 open reading frame 216
Details	13	97	hsa-miR-603	CEP135	centrosomal protein 135kDa
Details	14	96	hsa-miR-603	NPTX1	neuronal pentraxin I
Details	15	96	hsa-miR-603	SORL1	sortilin-related receptor, L(DLR class) A repeats containing
Details	16	96	hsa-miR-603	MMS22L	MMS22-like, DNA repair protein
Details	17	96	hsa-miR-603	KCNK10	potassium channel, subfamily K, member 10
Details	18	96	hsa-miR-603	STRBP	spermatid perinuclear RNA binding protein
Details	19	96	hsa-miR-603	FAM212B	family with sequence similarity 212, member B

Details	20	95	hsa-miR-603	PDS5B	PDS5, regulator of cohesion maintenance, homolog B (S. cerevisiae)
Details	21	95	hsa-miR-603	SLC35B4	solute carrier family 35, member B4
Details	22	95	hsa-miR-603	ADCY5	adenylate cyclase 5
Details	23	94	hsa-miR-603	PRKCA	protein kinase C, alpha
Details	24	94	hsa-miR-603	GIMAP8	GTPase, IMAP family member 8
Details	25	94	hsa-miR-603	MCTS1	malignant T cell amplified sequence 1
Details	26	94	hsa-miR-603	JAKMIP3	Janus kinase and microtubule interacting protein 3
Details	27	94	hsa-miR-603	HLF	hepatic leukemia factor
Details	28	93	hsa-miR-603	DSTYK	dual serine/threonine and tyrosine protein kinase
Details	29	93	hsa-miR-603	PTGFRN	prostaglandin F2 receptor negative regulator
Details	30	93	hsa-miR-603	KCNJ12	potassium inwardly-rectifying channel, subfamily J, member 12
Details	31	93	hsa-miR-603	PHF21A	PHD finger protein 21A
Details	32	93	hsa-miR-603	CDK6	cyclin-dependent kinase 6
Details	33	93	hsa-miR-603	SRCIN1	SRC kinase signaling inhibitor 1
Details	34	92	hsa-miR-603	MMAB	methylmalonic aciduria (cobalamin deficiency) cblB type
Details	35	92	hsa-miR-603	PCNX	pecanex homolog (Drosophila)
Details	36	92	hsa-miR-603	NDUFA4	NADH dehydrogenase (ubiquinone) 1 alpha subcomplex, 4, 9kDa
Details	37	92	hsa-miR-603	LGSN	lengsin, lens protein with glutamine synthetase domain
Details	38	92	hsa-miR-603	MGAT4A	mannosyl (alpha-1,3-)-glycoprotein beta-1,4-N-

					acetylglucosaminyltransferase, isozyme A
Details	39	92	hsa-miR-603	GSPT1	G1 to S phase transition 1
Details	40	91	hsa-miR-603	ATP9A	ATPase, class II, type 9A
Details	41	90	hsa-miR-603	SCAF11	SR-related CTD-associated factor 11
Details	42	90	hsa-miR-603	TM9SF3	transmembrane 9 superfamily member 3
Details	43	90	hsa-miR-603	ZNF670	zinc finger protein 670
Details	44	90	hsa-miR-603	ENPP4	ectonucleotide pyrophosphatase/phosphodiesterase 4 (putative)
Details	45	90	hsa-miR-603	ZNF117	zinc finger protein 117
Details	46	90	hsa-miR-603	HPS4	Hermansky-Pudlak syndrome 4
Details	47	89	hsa-miR-603	TRPM8	transient receptor potential cation channel, subfamily M, member 8
Details	48	89	hsa-miR-603	HIF1AN	hypoxia inducible factor 1, alpha subunit inhibitor
Details	49	89	hsa-miR-603	TFAP2C	transcription factor AP-2 gamma (activating enhancer binding protein 2 gamma)
Details	50	89	hsa-miR-603	FAM178A	family with sequence similarity 178, member A
